# Bibliographical

**Published:** 1863-12

**Authors:** 


### BIBLIOGRAPHICAL.

### The Principles and Practice of Dental Surgery.

By Chapin A. Harris, M. D., D. D. S.

Late President of the Baltimore College of Dental Surgery; Member of the American Medical Association; Author of Dictionary of Medical Terminology and Dental Surgery, ect., etc., etc.

Eighth Edition—Enlarged and revised—with 320 Illustrations.

Philadelphia—Lindsay & Blakiston—1863.

The publishers, in their preface to this edition, say—That in preparing this, the first posthumous edition of the late President Harris' Principles and Practice of Dental Surgery, they have spared no pains to make it in every way worthy of its own high reputation, and that of its distinguished Author.

The work has been thoroughly revised throughout, and to a large extent brought up with the times. Some important additions have been made to the work,—for instance, a full and complete description of rubber work, in all its applications to dental purposes; also a full presentation of the recent improvements in the treatment of cleft palate, together with many other particulars. This work has been accomplished by men eminently qualified for the task—by men who were intimately acquainted with Prof. Harris, and understood well his plans and desires in regard to his works; so that had he lived and made the revision himself, it would most probably have been just what it is.

So extensive have been the revisions and additions, that no former edition should exclude this from any dentist's library.

To the physician and surgeon, it would be a valuable acquisition. It is gotten up in Lindsay & Blakiston's best style, and is for sale, we presume, by most booksellers throughout the country. T.

